# Stability of vertical magnetic chains

**DOI:** 10.1098/rspa.2016.0703

**Published:** 2017-02

**Authors:** Johannes Schönke, Eliot Fried

**Affiliations:** Okinawa Institute of Science and Technology Graduate University, Onna, Okinawa 904-0495, Japan

**Keywords:** constrained mechanics, many body, non-local, athermal, buckling, bifurcation

## Abstract

A linear stability analysis is performed for a pair of coaxial vertical chains made from permanently magnetized balls under the influence of gravity. While one chain rises from the ground, the other hangs from above, with the remaining ends separated by a gap of prescribed length. Various boundary conditions are considered, as are situations in which the magnetic dipole moments in the two chains are parallel or antiparallel. The case of a single chain attached to the ground is also discussed. The stability of the system is examined with respect to three quantities: the number of balls in each chain, the length of the gap between the chains, and a single dimensionless parameter which embodies the competition between magnetic and gravitational forces. Asymptotic scaling laws involving these parameters are provided. The Hessian matrix is computed in exact form, allowing the critical parameter values at which the system loses stability and the respective eigenmodes to be determined up to machine precision. A comparison with simple experiments for a single chain attached to the ground shows good agreement.

## 1. Introduction

**Figure 1.**
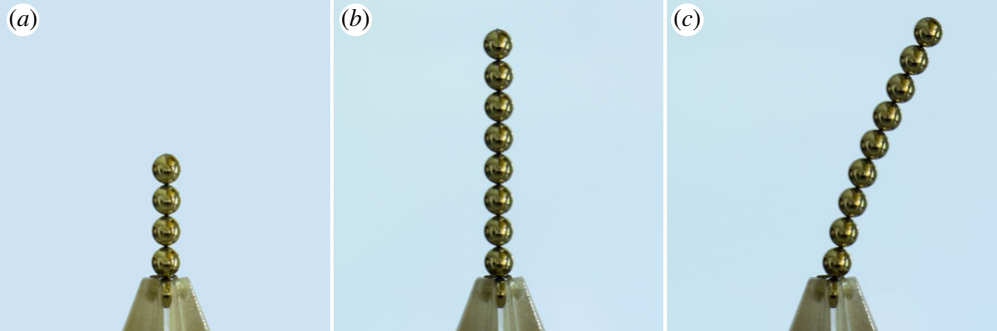
Stability of chains made from magnetic balls of diameter 5 mm, mass 0.5 g and magnetic flux density 1.19 T. A stack of no more than nine balls is stable (*a*,*b*) but a stack of 10 balls buckles under its own weight (*c*).

Permanently magnetized spherical balls provide fascinating avenues for studying many fundamental phenomena in physics. Being millimetre to centimetre-sized, they can be assembled by hand to form very complex structures. A simple but important question concerns the mechanical stability of chains formed from these magnetic balls: What is the maximum length of a chain of balls that can be balanced vertically without buckling by fixing the position and dipole orientation of its lowest element? For instance, although this is easily achieved with a chain of five commercially available toy balls of diameter 5 mm, mass 0.5 g and magnetic flux density 1.19 T, it is impossible with a chain of 10 such balls, as shown in figure 1! How does the maximum length of a chain of balls that can be balanced vertically without buckling depend on the diameter, mass and magnetic flux density of the constituent balls? Definitive answers to this and other related questions are provided in this paper.

It is a rare opportunity in modern physics to encounter a macroscopic, globally interacting many-body system which can be manipulated directly in our hands, watching and feeling its response, while at the same time having access to an accurate yet simple theory that encompasses its behaviour. In this paper, we present a rigorous mathematical framework to study the stability of many-body constrained mechanics including global interactions. Although we apply this framework only to chains of magnetic balls, it can readily be used for more complex assemblies such as sheets, as we discuss in §8. With reference to the observation, due to Vandewalle & Dorbolo [1], that for many-body *athermal* systems ‘… the interplay between small-scale physics and global properties is still a mystery’, we contribute here to this barely explored field. For athermal dipolar systems, phenomena that remain either completely or only partially understood include pattern formation in self-assembled structures (for example, in granular dipolar media under vibrations or external fields), the importance of metastable states, defects, frustration memory, structural stability and analogies to classical elastic materials. Our work can be viewed as a step towards increasing the understanding of such phenomena.

In one of the earliest studies of the collective behaviour of spherical dipoles, Weis & Levesque [2] found that such particles exhibit a strong tendency to form chains. In subsequent works, Clarke & Patey [3] and Messina *et al.* [4] found, depending on the number of particles, that the ground state is given by a sequence of straight chains, rings and tubes. In this regard, it is important to recognize that insofar as the dipole orientations are concerned, a tube formed by bending a sheet of spherical dipoles into a cylinder is equivalent to a tube formed by stacking appropriately sized rings.

Since chains provide a fundamental building block for many structures made from magnetized balls, it is natural to seek information concerning their effective elastic properties. Magnetic interactions make it energetically costly to bend such chains. In colloidal science, effects associated with dipolar particles have been explored in considerable detail (as evidenced by the review of Teixeira *et al.* [5]) and studies of the elastic behaviour of chains made from such particles have been conducted by Biswal & Gast [6], Cerda *et al.* [7] and Coq *et al.* [8]. The elastic properties of chains comprised by bacteria that contain magnetic nanoparticles have been studied by Kiani *et al.* [9].

Chains of magnetic toy balls provide alternative macroscopic systems for investigation. Vandewalle & Dorbolo [1] showed that mechanically stable defects can result from removing the central branch of a triple junction of chains made from these balls. Questions about the elastic properties of chains made from such balls were first addressed by Vella *et al.* [10] and Hall *et al.* [11], who introduced and demonstrated the utility of the notion of an effective magnetic bending stiffness. The first analysis of the buckling of a vertical magnetic chain under its own weight in a gravitational field was performed by Vella *et al.* [10]. Moreover, Hall *et al.* [11] used a discrete-to-continuum asymptotic analysis to derive an expression for the magnetic energy of a chain with local radius of curvature much larger than the diameter of the constituent balls. This result was used to determine the spectrum for the vibrations of a circular ring of spherical magnets, from which it was demonstrated that the small-amplitude oscillations of a ring of magnetic balls are effectively identical to those of an equivalent elastic ring made from an inextensible filament. In a related work, Boisson *et al.* [12] considered the dynamics of a horizontal chain of diametrically magnetized cylinders. Restricting attention to situations where neighbouring cylinders can only roll over one another without sliding, these authors determined the eigenfrequencies and associated eigenmodes arising from solving the relevant linearized equations of motion and found a close correspondence between the behaviour of their magnetic system and an elastic beam.

In this paper, we study the stability of vertical chains of magnetic balls under the influence of gravity. Instead of considering a single chain, we consider a system of two coaxial chains, one being attached to the ground and the other hanging from above with a gap of prescribed length. There are actually four different pathways along which this double-chain system can become unstable. Aside from the buckling of the lower chain due to gravity, the upper chain can rupture between its two uppermost balls as a consequence of the combined influences of gravity and the magnetic pull of the lower chain. If the lower chain is not fixed at its base, it can lose contact with the ground due to the magnetic pull of the upper chain. Finally, if the chains have opposing dipole orientations, then they can both buckle due to mutual repulsion. Two classes of boundary conditions are considered. For the clamped case, the position and dipole orientation of the ball next to the boundary is fixed. For the free case, the ball closest to the boundary is free to move tangentially without detaching from the boundary, and the dipole orientation is free. In our model, the particular problem of a single chain fixed at its base is recovered by allowing the gap to become infinite. We employ a linearized stability analysis to obtain explicit stability conditions in terms of eigenvalues and associated eigenmodes. Our methods allow us to evaluate these results to machine precision. We also provide new insights to the analogy between magnetic chains and classical elastic rods.

After introducing the model and the linear stability analysis in §2 and §3, we discuss four examples: the single clamped chain (§4), two equal clamped chains (§5), two equal free chains (§6) and two clamped chains with opposing dipole orientations (§7). The conclusions appear in §8.

## 2. Constrained systems of magnetic balls

We consider a finite system of identical magnetic balls—each of which is of diameter *d* and has spatially uniform mass density *ρ* and spatially uniform magnetic flux density *B*—in a spatially uniform gravitational field.

On denoting the centre position of ball *i* by **p**_*i*_, the vector **r**_*i*,*j*_ directed from the centre position of ball *j* to the centre position of ball *i* is given by

2.1 $${\text{r}}_{i,j}:={\text{p}}_{i}-{\text{p}}_{j},$$

with *r*_*i*,*j*_:=|**r**_*i*,*j*_| being the associated distance. Since each ball *i* has a spatially uniform magnetic flux density, its outer magnetic field is that of a point dipole and is fully specified by the magnetic dipole moment **m**_*i*_. The total potential energy *U* of the system incorporates the dipole–dipole interactions between each pair of balls and the influence of the external gravitational field. On introducing the permeability of free space *μ*_0_ and the mass *M*=*πρd*^3^/6 of a generic ball, this energy is given by

2.2 $$U=\frac{\mu_{0}}{4\pi }\sum_{i<j}\frac{\left({\text{m}}_{i}\cdot {\text{m}}_{j})r_{i,j}^{2}-3({\text{m}}_{i}\cdot {\text{r}}_{i,j})({\text{m}}_{j}\cdot {\text{r}}_{i,j}\right)}{r_{i,j}^{5}}+\text{Mg}\;{\text{e}}_{2}\cdot \sum_{i}{\text{p}}_{i},$$

where **e**_2_ denotes the unit vector directed upward antiparallel to the gravitational acceleration, which is of magnitude *g*>0.

It is convenient to work in a dimensionless setting where spatial distances are measured relative to *d*, magnetic dipole moments are measured relative to *m*:=*πBd*^3^/6*μ*_0_, and energies are measured relative to *Mgd*. Specifically, we define dimensionless counterparts **w**_*i*_, **x**_*i*,*j*_, *x*_*i*,*j*_, and **n**_*i*_, of **p**_*i*_, **r**_*i*,*j*_, *r*_*i*,*j*_, and **m**_*i*_ by

2.3 $${\text{w}}_{i}:=\frac{{\text{p}}_{i}}{d},\;{\text{x}}_{i,j}:=\frac{{\text{r}}_{i,j}}{d},\;x_{i,j}:=|{\text{x}}_{i,j}|\;\text{and}\;{\text{n}}_{i}:=\frac{{\text{m}}_{i}}{m},$$

respectively. Moreover, we define the dimensionless counterpart *E* of the energy *U* by

2.4 $$E:=\frac{\alpha }{6}\sum_{i<j}\frac{\left({\text{n}}_{i}\cdot {\text{n}}_{j})x_{i,j}^{2}-3({\text{n}}_{i}\cdot {\text{x}}_{i,j})({\text{n}}_{j}\cdot {\text{x}}_{i,j}\right)}{x_{i,j}^{5}}+{\text{e}}_{2}\cdot \sum_{i}{\text{w}}_{i},$$

where the dimensionless parameter

2.5 $$\alpha :=\frac{B^{2}}{4\mu_{0}\rho gd}>0$$

incorporates the competition between magnetic and gravitational forces.

For adjacent balls *i* and *j* to remain in contact, **x**_*i*,*j*_ must satisfy the constraint

2.6 $$x_{i,j}^{2}=1.$$

Additionally, since the magnitude of the dipole moment of each ball *i* is constant, the associated orientation **n**_*i*_ must satisfy the constraint

2.7 $$|{\text{n}}_{i}{|}^{2}=1.$$

To incorporate these constraints, we work with an augmented (dimensionless) potential energy of the form

2.8 $$F:=E+\sum_{x_{i,j}=1}\lambda_{i,j}(x_{i,j}^{2}-1)+\sum_{i}\nu_{i}(|{\text{n}}_{i}{|}^{2}-1),$$

and *λ*_*i*,*j*_ and *ν*_*i*_ are Lagrange multipliers associated, respectively, with the constraints (2.6) and (2.7).

Bearing in mind that **x**_*i*,*j*_ and **n**_*i*_ can be varied independently, we observe the system is in equilibrium only if the condition

2.9 $$\frac{\partial F}{\partial {\text{x}}_{i,j}}=\frac{\partial F}{\partial {\text{w}}_{i}}-\frac{\partial F}{\partial {\text{w}}_{j}}=\text{0}$$

holds for each pair *i* and *j* of balls that are adjacent and in contact and the condition

2.10 $$\frac{\partial F}{\partial {\text{n}}_{i}}=\text{0}$$

holds for each ball *i*.

## 3. System of two coaxial vertical magnetic chains separated by a finite gap

**Figure 2.**
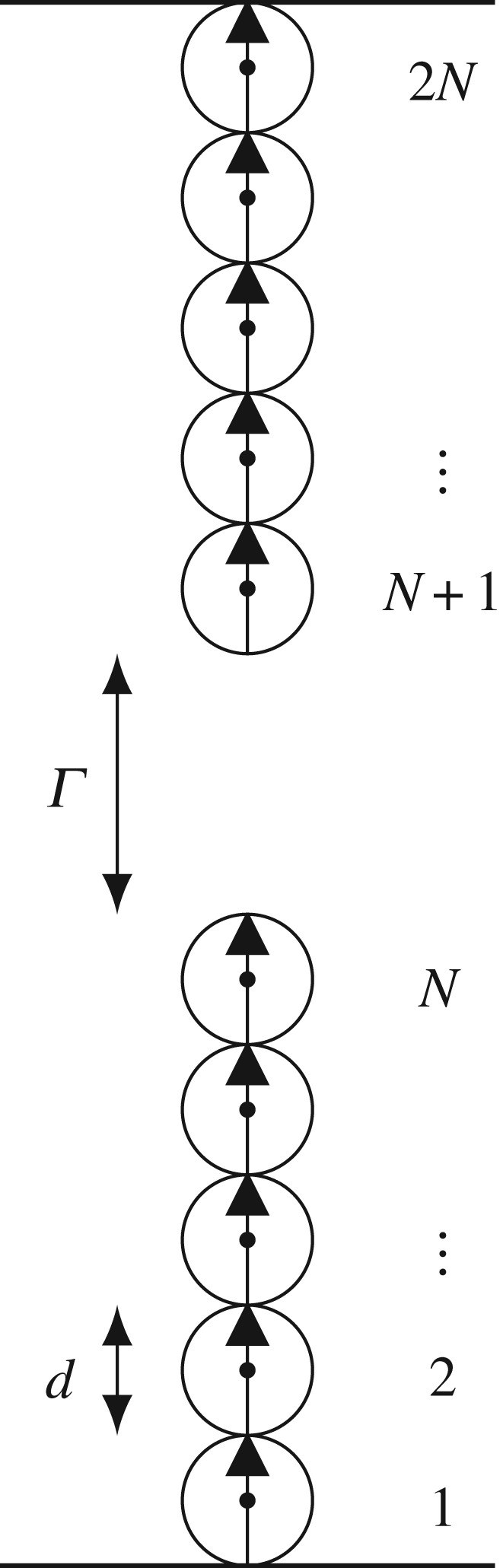
Two coaxial vertical magnetic chains with an equal number of balls.

We consider a system of two identical coaxial vertical chains each of them having *N* magnetic balls of diameter *d* separated by a distance *Γ*, as depicted in figure 2. It is easily confirmed that this configuration is an equilibrium configuration. The (dimensionless) gap is denoted by

3.1 $$\gamma :=\frac{\Gamma }{d}.$$

Until further notice, we assume that (i) the balls at the base of the lower chain and the apex of the upper chain must remain in contact with the adjacent surfaces, (ii) the balls comprising each chain remain in contact, and (iii) the dipole orientation of each ball is directed upward. We will eventually relax these assumptions to allow for (i) rupture of the upper chain at the junction between the two balls closest to its apex, (ii) lift off of the lower chain, and (iii) the dipole orientations of the upper chain to be directed downward and opposite to those in the lower chain. Note that reversing the orientation of all magnetic dipole moments leaves the energy unchanged and results in an equivalent state. We refer to this property as the polarity symmetry.

### (a) Lagrange multipliers

The 2(*N*−1) Lagrange multipliers *λ*_*i*,*i*+1_, *i*=1,…,*N*−1,*N*+1,…,2*N*−1, can be interpreted as the (dimensionless) reactive forces needed to ensure that immediately adjacent pairs of balls in the lower and upper chains remain in contact. These multipliers must satisfy the linear system

3.2 $$\left(\frac{\partial F}{\partial {\text{w}}_{i}}-\frac{\partial F}{\partial {\text{w}}_{i+1}}\right)\cdot {\text{x}}_{i,i+1}=0,\;i=1,\ldots ,N-1,N+1,\ldots ,2N-1,$$

which has a tridiagonal banded coefficient matrix. The solution to that system can be expressed as

3.3 $$\lambda_{i,i+1}=\left\{ \begin{array}{rl} & -\frac{\alpha }{4}\sum_{k=i}^{N-1}\left({\text{z}}_{k+1}\cdot {\text{x}}_{k,k+1}+\sum_{l=2}^{k}[{\text{z}}_{l}\cdot ({\text{x}}_{l-1,l}-{\text{x}}_{l,l+1})]\right)\\ & \;+\frac{1}{2}(N-i){\text{e}}_{2}\cdot {\text{x}}_{1,2},\;i=1,2,\ldots ,N-1,\\ & \frac{\alpha }{4}\sum_{k=N+2}^{3N+1-i}\left({\text{z}}_{k+1}\cdot {\text{x}}_{k,k+1}+\sum_{l=k}^{2N-1}[{\text{z}}_{l}\cdot ({\text{x}}_{l-1,l}-{\text{x}}_{l,l+1})]\right)\\ & \;+\frac{1}{2}(N-i){\text{e}}_{2}\cdot {\text{x}}_{2N-1,2N},\;i=N+1,\ldots ,2N-1, \end{array}\right.$$

where we have used the convention that $\sum_{i=a}^{b}\cdots =0$ if *b*<*a* and we have introduced

3.4 $${\text{z}}_{i}:=\sum_{j\neq i}\frac{1}{x_{i,j}^{5}}\left(({\text{n}}_{i}\cdot {\text{x}}_{i,j}){\text{n}}_{j}+({\text{n}}_{j}\cdot {\text{x}}_{i,j}){\text{n}}_{i}+\left[{\text{n}}_{i}\cdot {\text{n}}_{j}-\frac{5}{x_{i,j}^{2}}({\text{n}}_{i}\cdot {\text{x}}_{i,j})({\text{n}}_{j}\cdot {\text{x}}_{i,j})\right]{\text{x}}_{i,j}\right).$$

The remaining 2*N* Lagrange multipliers *ν*_*i*_, *i*=1,…,2*N*, can be interpreted as the reactive couples needed to ensure that the magnetic moment of each ball is of constant magnitude. These multipliers must satisfy the linear system

3.5 $${\text{n}}_{i}\cdot \frac{\partial F}{\partial {\text{n}}_{i}}=0,\;i=1,\ldots ,2N,$$

which can be solved to yield

3.6 $$\nu_{i}=\frac{\alpha }{12}\sum_{j,j\neq i}\frac{3({\text{n}}_{i}\cdot {\text{x}}_{i,j})({\text{n}}_{j}\cdot {\text{x}}_{i,j})-({\text{n}}_{i}\cdot {\text{n}}_{j})r_{i,j}^{2}}{r_{i,j}^{5}}.$$

### (b) Symmetries: planar version of the problem

It is evident that the system exhibits a rotational symmetry around the central vertical axis. Beyond that symmetry, an important question concerns the possible shapes of the first unstable mode arising from the stability analysis. Is that mode two-dimensional, so that the chains lie in a plane? Or is it three-dimensional, a helix for example? If we introduce cylindrical polar coordinates with central axis chosen to coincide with the axis shared by the undistorted chains and express the position **w**_*i*_ of each ball *i*=1,…,2*N* with respect to those coordinates, we find that, for any perturbations of the system, the gravitational potential energy is independent of the azimuthal angle. By contrast, the magnetic interactions energetically favour shapes in which all particle positions have the same azimuthal angle because such shapes have lower total curvatures. The first unstable mode must therefore lie in a plane that passes through the central axis of the coordinate system. In combination with the aforementioned rotational symmetry, it therefore suffices to restrict attention to perturbations involving only planar modes.

### (c) Structure of the Hessian

Consistent with the discussion in the previous section, we consider modes that lie in a plane characterized by a positively oriented orthonormal basis {**e**_1_,**e**_2_}, where **e**_2_ introduced in (2.2) is antiparallel to the gravitational acceleration. The constraints (2.6) requiring that balls in contact remain in contact dictate that the equilibrium position **w**_*i*_ of each ball *i* has at most a single translational degree of freedom in the direction of the horizontal basis element **e**_1_ and thus admits a representation of the form

3.7 $${\text{w}}_{i}=\left\{ \begin{array}{ll} t_{i}{\text{e}}_{1}+i{\text{e}}_{2}, & i=1,\ldots ,N,\\ t_{i}{\text{e}}_{1}+(i+\gamma ){\text{e}}_{2}, & i=N+1,\ldots ,2N, \end{array}\right.$$

where *t*_*i*_, *i*=1,…,*N*−1,*N*+1,…,2*N*−1, must obey

3.8 $$|t_{i}|\ll 1.$$

In view of (3.8), the difference

3.9 $${\text{x}}_{i+1,i}=(t_{i+1}-t_{i}){\text{e}}_{1}+{\text{e}}_{2}$$

describes an infinitesimal rotation of the axis between two immediately adjacent balls *i* and *i*+1 for each *i*=1,…,*N*−1,*N*+1,…,2*N*−1.

The remaining constraints (2.7) dictate that the equilibrium magnetic moment **n**_*i*_ of each ball *i* has at most a single degree of freedom in the direction of the horizontal basis element **e**_1_, describing rotation of the moment in the plane, and thus admits a representation of the form

3.10 $${\text{n}}_{i}=s_{i}{\text{e}}_{1}+{\text{e}}_{2},$$

where *s*_*i*_, *i*=1,…,2*N*, must obey

3.11 $$|s_{i}|\ll 1.$$

In the view of (3.11), **n**_*i*_ as given by (3.10) describes an infinitesimal rotation of the magnetic moment of ball *i*.

In terms of the column vectors

3.12 $$\text{t}=\left(\begin{array}{c} t_{1}\\ \vdots \\ t_{2N} \end{array}\right)\;\text{and}\;\text{s}=\left(\begin{array}{c} s_{1}\\ \vdots \\ s_{2N} \end{array}\right),$$

the base configuration in which both chains are straight and coaxial is captured by setting **t**=**s**=**0**. For perturbations of the form (3.7) and (3.10), evaluating the Hessian of (2.8) at the base configuration yields a symmetric 4*N*×4*N* matrix

3.13 $$\text{H}=\left(\begin{array}{cc} {\text{H}}_{tt} & {\text{H}}_{ts}\\ {\text{H}}_{st} & {\text{H}}_{ss} \end{array}\right),$$

where the (*i*,*j*)th entries of the blocks **H**_*tt*_, **H**_*ts*_=**H**_*st*_ and **H**_*tt*_ are given by

3.14 $${\frac{\partial^{2}F}{\partial t_{i}\partial t_{j}}|}_{\text{t}=\text{s}=\text{0}},\;{\frac{\partial^{2}F}{\partial t_{i}\partial s_{j}}|}_{\text{t}=\text{s}=\text{0}}={\frac{\partial^{2}F}{\partial s_{i}\partial t_{j}}|}_{\text{t}=\text{s}=\text{0}}\;\text{and}\;{\frac{\partial^{2}F}{\partial s_{i}\partial s_{j}}|}_{\text{t}=\text{s}=\text{0}},$$

respectively. The Hessian can, alternatively, be expressed as the sum

3.15 H=C+αA

of a purely gravitational component **C** and a purely magnetic component *α***A**.

Consistent with its gravitational nature, **C** is independent of the magnetic moments of the balls and thus has block structure

3.16 $$\text{C}=\left(\begin{array}{cc} {\text{C}}_{tt} & \text{0}\\ \text{0} & \text{0} \end{array}\right),$$

where **C**_*tt*_ is a block diagonal 2*N*×2*N* matrix of the form

3.17 $${\text{C}}_{tt}=\left(\begin{array}{cc} {\text{C}}_{tt}^{L} & \text{0}\\ \text{0} & {\text{C}}_{tt}^{U} \end{array}\right),$$

with ${\text{C}}_{tt}^{L}$ and ${\text{C}}_{tt}^{U}$ being symmetric and tridiagonal *N*×*N* matrices that stem from the action of gravity on the balls that, respectively, comprise the lower and upper chains. Whereas ${\text{C}}_{tt}^{L}$ has diagonal

3.18 $$\left\{-N+1,{\left(-2N+2i+1\right)}_{i=1}^{N-1}\right\}$$

and neighbouring diagonals

3.19 $$\{{\left(N-i\right)}_{i=1}^{N-1}\},$$

${\text{C}}_{tt}^{U}$ has diagonal

3.20 $$\left\{{\left(2N-2i-1\right)}_{i=1}^{N-1},N-1\right\}$$

and neighbouring diagonals

3.21 $$\{{\left(i-N\right)}_{i=1}^{N-1}\}.$$

With (3.18)–(3.21), a direct calculation shows that ${\text{C}}_{tt}^{L}$ and ${\text{C}}_{tt}^{U}$ are, respectively, negative- and positive-semidefinite. This is consistent with the observation that only the lower chain can undergo a buckling instability due to gravity.

The 2*N*×2*N* matrix **A** that determines the remaining term in the additive decomposition (3.15) of the Hessian **H** is generally a full 2*N*×2*N* matrix of the form

3.22 $$\text{A}={\text{A}}_{0}+\sum_{n=1}^{2N-1}\sum_{l=0}^{2}\frac{{\text{A}}_{ln}\gamma^{l}}{{\left(\gamma +n\right)}^{5}},$$

with **A**_0_ and **A**_*ln*_ being 6*N*−2 matrices consisting of rational entries that depend only on *N* for each combination of *l*=0,1,2 and *n*=1,…2*N*−1. However, a symbolic manipulation programme can be used to obtain and evaluate these entries up to machine precision. In the subsequent analysis, which rests on an eigenvalue decomposition of **H**, we use Mathematica for all symbolic calculations. Without providing a proof, we note that **A** is positive-semidefinite.

### (d) Boundary conditions

**Figure 3.**
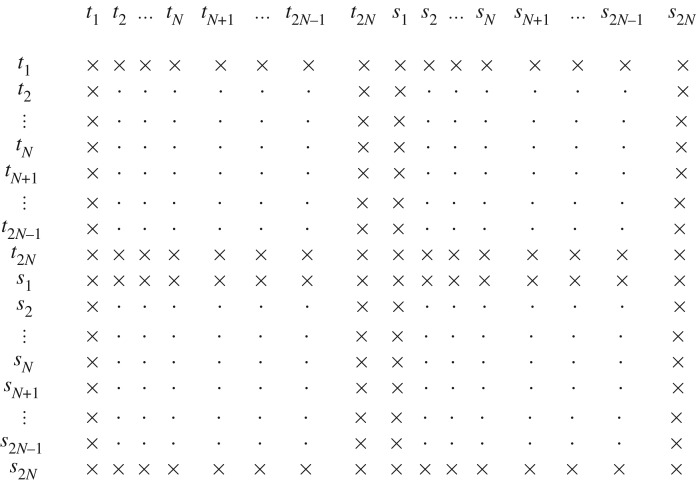
Schematic diagram depicting the removal (×) of rows and columns from **H** stemming from the imposition of certain boundary conditions.

So far, the system can explore all possible degrees of freedom in the sense that each ball i=1,…,2N can move horizontally and its dipole moment can rotate. A collective rigid translation of the entire system in the direction **e**_1_ is therefore possible. This translational symmetry is reflected in the degeneracy of **H**. However, fixing the horizontal position of one ball alleviates this difficulty. This can be achieved by fixing the horizontal positions of either ball *i*=1 or ball *i*=2*N* and, thus, can arise as a natural consequence of imposing certain boundary conditions. If, for instance, the horizontal positions and dipole moments of balls *i*=1 and *i*=2*N* are fixed, so that *t*_1_=*s*_1_=*t*_2*N*_=*s*_2*N*_=0, then the corresponding Hessian can be obtained by deleting the rows and columns for *t*_1_, *t*_2*N*_, *s*_1_, and *s*_2*N*_ from the general expression (3.13), as depicted in figure 3. Alternative boundary conditions are discussed subsequently.

### (e) Criteria for stability

The stability of a given configuration of the system is determined by the spectrum *η*_*i*_, *i*=1,…,*Q* of eigenvalues of the Hessian **H** evaluated at that configuration. The number *Q* of eigenvalues of **H** depends on the chosen boundary conditions. If *η*_*i*_>0 for all *i*=1,…,*Q*, then the configuration is stable. If *η*_*i*_<0 for one or more *i*=1,…,*Q*, then the configuration is unstable. If the smallest eigenvalue *η*_*i*_ of **H** vanishes for some value of a system parameter (such as *α* defined in (2.5)), then the configuration loses stability and the corresponding value of the parameter in question is referred to ‘critical’. This is an example of a bifurcation point. The system is said to undergo a bifurcation, and therefore change its stability properties, each time an eigenvalue *η*_*i*_ of **H** becomes zero. In the view of the representation $\det\text{H}=\eta_{1}\eta_{2}\cdots \eta_{Q}$, the bifurcation points correspond to the zeros of detH.

## 4. Stability of a chain clamped at its base

We first explore the stability of the lower chain in isolation, with the stipulation that it is clamped at its base. This situation can be achieved formally by taking the limit γ→∞ and neglecting the upper chain entirely. Under these circumstances, the stability of the lower chain involves only two parameters, namely the number *N* of balls it contains and the measure *α* of the competition between magnetic and gravitational forces. We assume that the lowermost ball *i*=1 of the lower chain is clamped in the sense that it can neither translate nor rotate. The Hessian of the base configuration then reduces to the 2(*N*−1)×2(*N*−1) matrix $\bar{\text{H}}$ that arises on removing the rows and columns for *t*_*i*_ and *s*_*i*_, *i*=1,*N*+1,…,2*N*, from (3.13). Moreover, on noting that the sum on the right-hand side of (3.22) vanishes in the limit γ→∞, the decomposition (3.15) specializes to an expression of the form

4.1 $$\bar{\text{H}}=\bar{\text{C}}+\alpha {\bar{\text{A}}}_{0},$$

where, with reference to (3.16) and (3.17), only the upper left block ${\bar{\text{C}}}_{tt}^{L}$ of $\bar{\text{C}}$, which is of rank *N*−1, is non-vanishing. Since the lowermost ball is clamped, the degeneracy discussed in §3d is removed and it follows that ${\bar{\text{C}}}_{tt}^{L}$ is negative-definite and **A**_0_ is positive-definite. It is crucial that the ball at the base of the lower chain is clamped. Otherwise, the system is unconditionally unstable.

It might seem natural to consider particular values of *α*—or, equivalently, particular combinations of the parameters entering the definition (2.5) of *α*—and then determine the associated critical number *N*_*c*_ of balls that can be placed on top of one another before the chain becomes unstable. However, since *N* is integer valued, *N*_*c*_ must be discontinuous. It is therefore advantageous to fix *N* and determine the associated critical value *α*_*c*_ of *α* below which the chain is unstable. Hence, *α*_*c*_ must be a bifurcation point of the Hessian $\bar{\text{H}}$, namely a point at which $\det\bar{\text{H}}=0$. To determine all bifurcation points of $\bar{\text{H}}$, we invoke the representation (4.1) and the invertibility of ${\bar{\text{A}}}_{0}$ to express the determinant of $\bar{\text{H}}$ in the form

4.2 $$\det\bar{\text{H}}=\det{\bar{\text{A}}}_{0}\det(\alpha {\text{I}}_{2(N-1)}+{\bar{\text{A}}}_{0}^{-1}\bar{\text{C}})=\alpha^{N-1}P_{N-1}(\alpha ),$$

where **I**_*K*_ denotes the *K*×*K* identity matrix and where

4.3 $$P(\alpha ):=\det{\bar{\text{A}}}_{0}\det[\alpha {\text{I}}_{N-1}+{\left({\bar{\text{A}}}_{0}^{-1}\right)}_{tt}{\bar{\text{C}}}_{tt}]$$

is a polynomial of order *N*−1 in *α*. Since *α*>0 and ${\bar{\text{A}}}_{0}$ is positive definite, we infer from (4.2) that

4.4 $$\det\bar{\text{H}}=0\;⟺\;P(\alpha )=0,$$

and, thus, that $\bar{\text{H}}$ has at most *N*−1 distinct bifurcation points corresponding to the roots of P(α)=0 or, by (4.3), the eigenvalues of the (*N*−1)×(*N*−1) matrix $-{\left({\bar{\text{A}}}_{0}^{-1}\right)}_{tt}{\bar{\text{C}}}_{tt}$. In appendix A, we establish the connection between the determinants in (4.2) and (4.3) and also show that the roots of P(α)=0 must always be real and positive.

If the roots of P(α) are labelled in ascending order, so that *α*_1_≤⋯≤*α*_*N*−1_, then the critical value *α*_*c*_ of *α* below which the chain is unstable must be given by

4.5 $$\alpha_{c}=\alpha_{N-1}.$$

Up to *N*=50, the eigendecomposition shows that only a single eigenmode exhibits an exchange of stability at each bifurcation point. Moreover, when regarded as functions of *α*, two distinct eigenvalues *η*_*i*_ and *η*_*j*_, *i*≠*j*, do not cross each other for the named range of *N*. In combination with (4.2) and (4.4), we can therefore conclude that $\bar{\text{H}}$ has one family of *N*−1 conditionally stable (eigen)modes, where each mode loses stability at an *α* value associated with one unique root of P(α)=0. Furthermore, there is a family of *N*−1 unconditionally stable modes which never destabilize for *α*>0. These two families of modes exist because the gravitational contribution to the energy (2.8) is independent of the orientations of the dipole moments. To see this connection, note that the factoring of *α*^*N*−1^ in $\det\bar{\text{H}}$ is only possible because of the *N*−1 rank deficiency in $\bar{\text{C}}$ (see appendix A). This rank deficiency arises simply because the gravitational energy is independent of the orientations of the dipole moments.

### (a) Stability threshold for a chain consisting of two balls

Despite its simplicity, the case of *N*=2 balls affords valuable insight. In this case,

4.6 $$\bar{\text{H}}=\bar{\text{C}}+\alpha {\bar{\text{A}}}_{0}=\left(\begin{array}{cc} -1 & 0\\ 0 & 0 \end{array}\right)+\frac{\alpha }{6}\left(\begin{array}{cc} 6 & -3\\ -3 & 2 \end{array}\right).$$

Calculating $-{\left({\bar{\text{A}}}_{0}^{-1}\right)}_{tt}{\bar{\text{C}}}_{tt}$, we find that P(α)=α−4 and, thus, that the critical value *α*_*c*_ of *α* is given by

4.7 $$\alpha_{c}^{N=2}=4.$$

Moreover, we find that $\bar{\text{H}}$ possesses a conditionally stable eigenvalue

4.8 $${\bar{\eta }}_{1}=\frac{4\alpha -3-q}{6}$$

with $q:=\sqrt{13\alpha^{2}-12\alpha +9}$, satisfying ${\bar{\eta }}_{1}<0$ for *α*<4 and ${\bar{\eta }}_{1}>0$ for *α*>4, along with an unconditionally stable eigenvalue

4.9 $${\bar{\eta }}_{2}=\frac{4\alpha -3+q}{6}$$

satisfying ${\bar{\eta }}_{2}>0$. Plots of these eigenvalues and depictions of the corresponding modes, which obey

4.10 $${\bar{\text{v}}}_{1}\propto \left(\begin{array}{c} 3-2\alpha +q\\ 3\alpha \end{array}\right)\;\text{and}\;{\bar{\text{v}}}_{2}\propto \left(\begin{array}{c} 3-2\alpha -q\\ 3\alpha \end{array}\right)$$

appear in figure 4.

**Figure 4.**
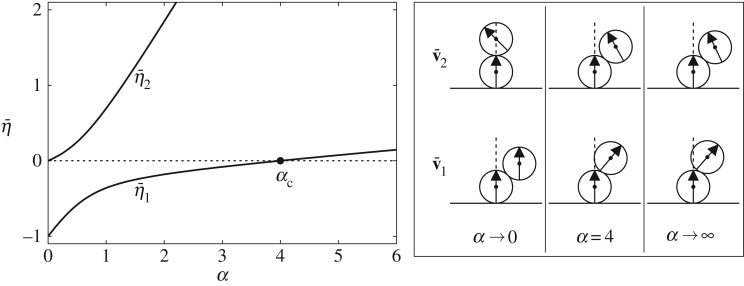
Eigenvalues ${\bar{\eta }}_{1}$ and ${\bar{\eta }}_{2}$ for the single clamped chain with two balls as functions of *α* and corresponding (eigen)modes ${\bar{\text{v}}}_{1}$ and ${\bar{\text{v}}}_{2}$ for *α*→0, *α*=4 and α→∞. For *α*<4, the base configuration is unstable against perturbations in the direction of mode ${\bar{\text{v}}}_{1}$.

Since our focus is on the stability of the base configuration, ${\bar{\text{v}}}_{1}$ and ${\bar{\text{v}}}_{2}$ should not be mistaken for new equilibrium shapes. Rather, they describe the directions in which an infinitesimal perturbation can be applied to the base configuration. The sign of the corresponding eigenvalue then dictates whether or not the base configuration is stable with respect to any infinitesimal perturbation in the direction of the associated mode.

### (b) General stability threshold: asymptotic scaling for long chains

**Figure 5.**
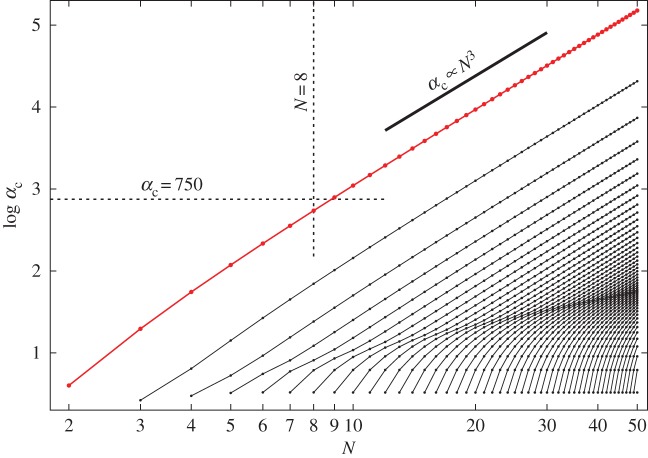
Critical value *α*_*c*_ of the dimensionless parameter *α* defined in (2.5) for the stability of the single clamped chain as a function of the number of balls *N* (red curve). Curves in black show the consecutive values of *α* corresponding to bifurcation points of the Hessian $\bar{\text{H}}$ (4.1) at which a mode higher than the first mode loses stability. For a given *N*, we have *N*−1 conditionally stable modes and therefore *N*−1 bifurcation points. The dashed black lines demonstrate that, for *α*=750 (which is the experimental value for the balls which appear in figures 1, 14 and 15), a chain consisting of no more than *N*=8 balls is stable. The thick black line segment shows the asymptotic slope of *α*_*c*_ as N→∞.

The magnetic forces must be increasingly strong relative to gravity to keep a chain clamped at its base from bending as the number *N* of balls in the chain increases. Consistent with this observation, the critical value *α*_*c*_ of *α* at which the base configuration loses stability increases monotonically with *N*. What is perhaps surprising is how rapidly this increase occurs. In particular, for a chain of three balls $\alpha_{c}^{N=3}=4(293+\sqrt{50009})/105\approx 19.7$, which represents a nearly 400% increase over the value $\alpha_{c}^{N=2}=4$ for a chain of two balls. Figure 5 contains a plot of *α*_*c*_ as a function of *N* together with various other non-vanishing values of *α* corresponding to bifurcations involving higher modes. Experiments conducted with balls of diameter 5 mm, mass 0.5 g and magnetic flux density 1.19 T demonstrate that a chain of *N*=8 such balls can be held straight without difficulty and that, with sufficient care, it is also possible to hold a chain of *N*=9 such balls straight. However, these experiments also demonstrate that it is impossible to hold a chain of *N*=10 such balls straight. Photographs for chains with 9 and 10 balls are shown in figure 1. For the given experimental parameters, definition (2.5) of *α* yields *α*≈750, which is slightly less restrictive than the theoretical prediction $\alpha_{c}^{N=9}\approx 790$ that can be read off from figure 5 but well above the next lowest prediction $\alpha_{c}^{N=8}\approx 544$. This small disparity between theory and experiment is likely due to the absence of frictional effects in our model.

Inspection of figure 5 shows that, for sufficiently large values of *N*, *α*_*c*_ scales with *N*^3^, as do the curves corresponding to bifurcations involving higher modes. Consistent with the discussion of Vella *et al.* [10], this scaling suggests that, in the continuum limit, a straight chain of magnetic balls behaves like a classical heavy elastica. Hall *et al.* [11] showed that the contributions to the energy of a chain of magnetic balls due to local and non-local magnetic interactions give rise to terms that also scale with *N*^3^ as N→∞. With this in mind, the local contribution to the energy calculated by Hall *et al.* [11] can be used to yield an effective bending stiffness

4.11 $$K_{loc}=\zeta (3)\kappa ,\;\kappa :=\frac{\pi B^{2}d^{4}}{288\mu_{0}}=\frac{Mgd^{2}\alpha }{12},$$

where $\zeta (3)=\sum_{i=1}^{\infty }i^{-3}\approx 1.202$. Then, following Vella *et al.* [10] and using (4.11) in conjunction with Wang's [13] refinement of Greenhill's [14] expression for the maximum height to which a heavy elastic standing column that is cantilevered at its base can be constructed without becoming unstable, we are led to the approximation

4.12 $$\alpha_{c}^{\infty }=kN^{3},$$

with the prefactor *k* being given by

**Figure 6.**
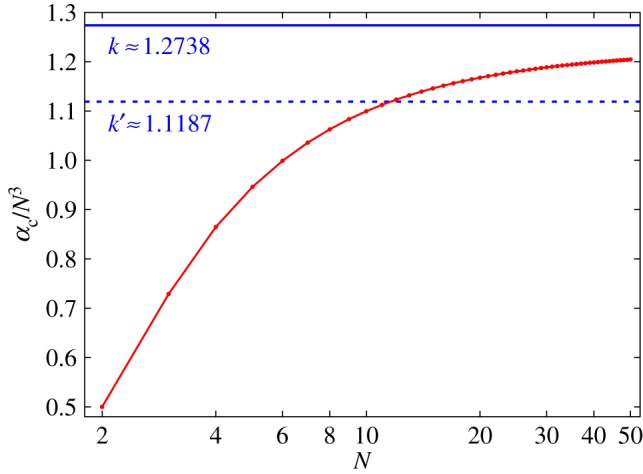
Critical value *α*_*c*_ (red curve) as in figure 5. The plot is scaled by *N*^3^. The solid blue line shows the critical asymptote $\alpha_{c}^{\infty }$ (4.12) for N→∞ with the prefactor *k* from (4.13) obtained under the assumption of only local magnetic interactions. The dashed blue line is an asymptote analogue to (4.12) with the prefactor *k*^′^ obtained from Vella *et al.* [10].

4.13 $$k=\frac{16}{3\zeta (3)z_{1}^{2}}\approx 1.2738,$$

where *z*_1_≈1.86635 is the first zero of the Bessel function of the first kind of order −1/3. The approximation (4.12)–(4.13) should become increasingly accurate in the continuum limit, namely as N→∞. The plots in figure 6 show that *α*_*c*_ approaches $\alpha_{c}^{\infty }$ asymptotically from below and, thus, that $\alpha_{c}^{\infty }$ provides a reasonable approximation to *α*_*c*_ for chains consisting of as few as *N*≈30 balls. We therefore refer to $\alpha_{c}^{\infty }$ as the ‘critical asymptote’.

In contrast to the generally valid local contribution (4.11) to the effective bending stiffness, the non-local contribution *K*_*nonloc*_ depends on the current shape of the chain. For a closed ring, this contribution leads to $K_{nonloc}^{ring}=\kappa /6$, as shown by Vella *et al.* [10] and Hall *et al.* [11]. Even though the resulting total effective bending stiffness, $K^{ring}=K_{loc}+K_{nonloc}^{ring}=(\zeta (3)+1/6)\kappa$, is exact only for the closed ring (see the final sentence of Vella *et al.* [10], §2(d)), Vella *et al.* [10] use it for the vertical clamped chain as well. With this assumption, Vella *et al.* [10], eqn. (3.2) obtain an estimate for the critical value *N*_*c*_ of *N* as N→∞. In combination with the relation

4.14 $$\alpha =\frac{B^{2}}{4\mu_{0}\rho gd}=\frac{1}{8G}$$

between *α* and the magneto-gravitational number G of Vella *et al.* [10], eqn. (3.3), that estimate leads to a relation of the form (4.12) but with a value *k*^′^≈1.1187 of the prefactor *k* slightly lower than that appearing in (4.13). Figure 6 shows that the corresponding approximation to *α*_*c*_ does not provide a valid asymptote because it crosses *α*_*c*_ between *N*=11 and *N*=12.

Using the relation, $N_{c}\approx 0.4613G^{-1/3}$, between *N*_*c*_ and G corresponding to (4.12) and (4.13) also leads to an improvement on the fit appearing in Fig. 5 of Vella *et al.* [10]. Since non-local interactions are neglected from the calculation leading to (4.12), this improvement raises a question about the extent to which the effective bending stiffness is sensitive to those interactions. Since the asymptotic analysis of Hall *et al.* [11] breaks down near the ends of a chain, a different strategy is needed to determine the non-local contribution to the effective bending stiffness. We expect several effects to be operative. One of these stems from the non-local interactions away from the boundaries and would yield a non-local term corresponding to that arising in the work of Hall *et al.* [11], eqn. (3.51). If, however, this were the only non-local effect, we would certainly obtain $K_{nonloc}^{straight\;chain}<K_{nonloc}^{ring}=\kappa /6$, because otherwise, as mentioned above, the relation *α*_*c*_=*kN*^3^ would intersect our theoretical estimate and, thus, would no longer provide an asymptote for large *N*. Another non-local effect stems from the endpoints of the chain, which in the continuum limit act as oppositely charged monopoles. Since bending a straight chain brings these attractive poles closer together and therefore decreases the energy, this effect lowers the non-local bending stiffness. The boundary conditions (such as the clamping of the chain at its base) would also exert an influence. The problem of determining the exact form of the prefactor entering the critical asymptote is an interesting problem but is beyond the scope of this paper. The results reported here nevertheless support the findings of Hall *et al.* [11] and Vella *et al.* [10] showing that, for infinitesimal strains, it is reasonable to describe a chain of magnetic balls as an elastic rod.

### (c) Sample modes for a chain consisting of eight balls

**Figure 7.**
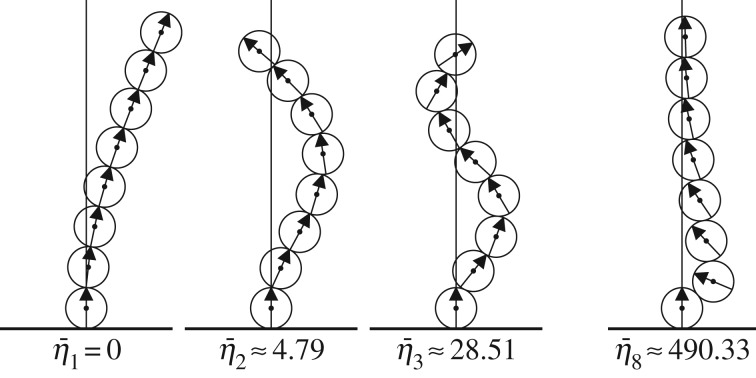
The first three modes ${\bar{\text{v}}}_{1}$, ${\bar{\text{v}}}_{2}$ and ${\bar{\text{v}}}_{3}$ and the first unconditionally stable mode ${\bar{\text{v}}}_{8}$ for the single clamped chain of eight balls at the critical $\alpha_{c}^{N=8}\approx 544$ where the first mode destabilizes. The corresponding eigenvalue is indicated below each mode.

For a chain of *N*=8 balls with $\alpha =\alpha_{c}^{N=8}\approx 544$, several different modes are depicted in figure 7. Being at the critical *α* value, the first mode destabilizes at this point and the corresponding eigenvalue vanishes. The shapes of the first three modes are reminiscent of bending modes for a classical heavy elastic rod, which is reasonable in view of the discussion in the previous paragraph.

Figure 7 also shows the eighth mode (out of 14 modes in total), which is the first unconditionally stable mode. The nature of this mode differs fundamentally from the lower bending modes. The second ball (counting upward from the base) performs a strong sliding motion and the uppermost ball moves essentially longitudinally.

## 5. Two clamped chains separated by a finite gap

We now return to the original problem involving two chains separated by a gap *γ* that is both positive and finite, with the stipulation that the lower chain is clamped at its base and the upper chain is clamped at its apex. For sufficient small values of *γ*, we intuitively expect that magnetic interactions with the upper chain should stabilize the lower chain. Additionally, however, the magnetic force exerted on the upper chain by the lower chain can lead to rupture of the upper chain. We explore this possibility before considering the problem of stability in the absence of rupture.

### (a) Rupture of the upper chain

Although we formulated the problem under the assumption that any pair of balls which are in contact in the base configuration remain in contact, it is important to recognize that the magnetic interactions that maintain the integrity of the upper chain must compete with the downward forces exerted by gravity and by magnetic interactions with the balls of the lower chain.

Since the upper chain is clamped at its apex, we expect that any rupture must occur at the junction between the clamped ball *i*=2*N* at its apex and its immediate neighbour, ball *i*=2*N*−1.

#### (i) Rupture due solely to gravity

We first consider the upper chain in isolation, which can be achieved formally by taking the limit γ→∞ and neglecting the lower chain entirely. In this context, the threshold for rupture is reached when the magnitude of the attractive magnetic force between the ball at the apex of the upper chain and its other *N*−1 balls is balanced by the gravitational force acting on those remaining balls. In dimensionless form, this criterion yields a relation between *N* and *α*:

5.1 $$\alpha \sum_{i=1}^{N-1}\frac{1}{i^{4}}=N-1.$$

Since the per cent error between the partial sum $\sum_{i=1}^{N-1}i^{-4}$ and its infinite counterpart $\zeta (4)=\sum_{i=1}^{\infty }i^{-4}=\pi^{4}/90$ decreases monotonically with *N* and is below 1% for *N*≥4, (5.1) yields, to good approximation, an expression

5.2 $$N_{r}=1+\frac{\pi^{4}\alpha }{90}$$

for the critical number of balls at which rupture occurs. Thus, *N*_*r*_ grows linearly with *α*. In combination, the definition (2.5) of *α* and (5.2) give rise to an expression for the critical length

5.3 $$\ell_{r}:=N_{r}d=d+\frac{\pi^{4}B^{2}}{360\mu_{0}\rho g}$$

of a chain on the threshold of rupture. Granted that *α*≫1, (5.2) can be approximated by *N*_*r*_=*π*^4^*α*/90 and (5.3) leads us to infer that the critical length ℓ_*r*_ at which a vertically hanging chain clamped at its apex ruptures is independent of the size of the balls from which it is made.

#### (ii) General rupture

If the magnetic force exerted by the lower chain on the upper chain is taken into consideration, then the balance (5.1) between magnetic and gravitational forces generalizes to

5.4 $$\alpha \sum_{i=1}^{N-1}\left[\frac{1}{i^{4}}-\sum_{k=1}^{N}\frac{1}{{\left(i+k+\gamma -1\right)}^{4}}\right]=N-1.$$

Numerical methods can be used to extract from (5.4) a relation for the onset of rupture in which one of the three parameters *N*, *α* and *γ* is given in terms of the other two.

A relatively simple case arises in the limit α→∞ corresponding to situations where gravity is dominated by magnetic interactions. Consistent with intuition, it is evident from (5.4) that rupture induced by the attractive magnetic interactions between the chains can still occur in the absence of gravity. Increasing *N* incrementally, we find that the critical gap *γ*_*r*_ corresponding to the onset of rupture varies between $\gamma_{r}^{N=2}\approx 0.0157$ and $\gamma_{r}^{N\to \infty }\approx 0.0296$, which represents a relatively small range of gaps. Also consistent with intuition is the observation that, in the limit α→∞, the lower and upper chains become indistinguishable. Thus, rupture should occur not only in the upper chain but also mirror-symmetrically (with respect to the line through the midpoint of the gap and orthogonal to the chains) in the lower chain as well.

### (b) Stability of the base configuration

For clamped chains separated by a gap *γ*, the Hessian $\tilde{\text{H}}$ of the base configuration is now the 4(*N*−1)×4(*N*−1) matrix that results on removing the rows and columns of (3.15) as described in §3d and illustrated in figure 3. Proceeding as in the derivation of $\bar{\text{H}}$, we find that

5.5 $$\det\tilde{\text{H}}=:\alpha^{2(N-1)}P^{-}(\alpha ;\gamma )P^{+}(\alpha ;\gamma ),$$

where $P^{\pm }(\alpha ;\gamma )$ is a polynomial of order *N*−1 in *α* for each finite choice of *γ*. Whereas the roots $\alpha_{i}^{+}(\gamma )$, *i*=1,…,*N*−1, of $P^{+}$ are positive, the roots $\alpha_{i}^{-}(\gamma )$, *i*=1,…,*N*−1, of $P^{-}$ are negative. Thus, for each 0<γ<∞, there are at most *N*−1 bifurcation points $\alpha_{i}^{+}(\gamma )$, *i*=1,…,*N*−1, associated with the solutions to $P^{+}(\alpha ;\gamma )=0$. Therefore, there are *N*−1 conditionally stable modes but 3(*N*−1) unconditionally stable modes. If, as in the treatment of the problem involving a single chain, the roots of $P^{+}(\alpha ;\gamma )$ are arranged in ascending order, so that $\alpha_{1}^{+}\leq \cdots \leq \alpha_{N-1}^{+}$, then the critical value *α*_*c*_ of *α* below which the lower chain is unstable must be $\alpha_{N-1}^{+}$.

#### (i) Stability threshold for chains consisting of two balls

In contrast to what occurs for a single isolated chain consisting of *N*=2 balls, it is impractical to provide expressions for the eigenvalues and associated modes of $\tilde{\text{H}}$, at least for arbitrary choices of *α*>0 and 0<γ<∞. With some effort, however, $\alpha_{c}^{N=2}(\gamma )$ can be expressed in full generality (as shown in appendix B). For instance, for *γ*=1 (which corresponds to a gap length equal to the diameter of a single ball), we obtain a critical value $\alpha_{c}^{N=2}(1)\approx 1.784$ of *α* that is less than one-half of the corresponding value *α*_*c*_=4 for a single isolated chain clamped at its base. In general, the function $\alpha_{c}^{N=2}(\gamma )$ increases monotonically from $\alpha_{c}^{N=2}(\gamma \to 0)\approx 0.3166$ to the asymptotic value *α*_*c*_=4, with a steep initial rise; in particular, at *γ*=5 we find that $\alpha_{c}^{N=2}(5)\approx 3.8622$.

#### (ii) General stability threshold

**Figure 8.**
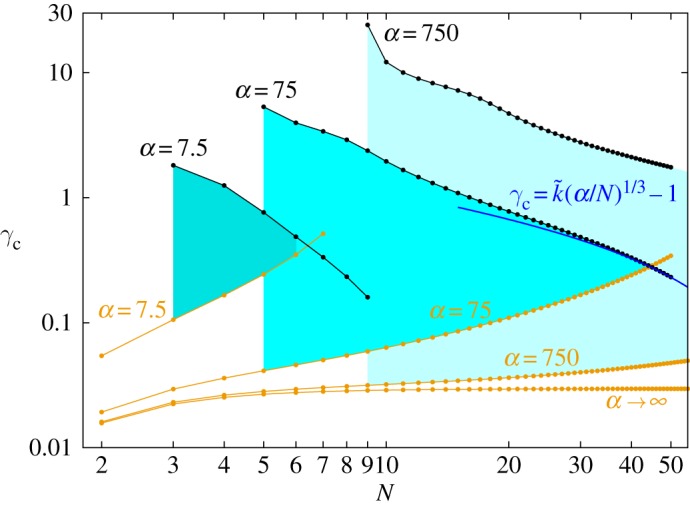
Critical gap *γ*_*c*_ (black) below which two equal clamped chains (each containing *N* balls) are stable. Several curves for different values of *α* are plotted. Orange lines show the *γ*_*r*_ below which the upper chain will rupture (§5a). Cyan areas indicate the allowed ranges for *N* and *γ* to have stable chains that do not rupture for a given *α*. The blue line exemplarily shows the asymptotic behaviour of *γ*_*c*_ as N→∞.

For each combination of *N* and *α*, it is generally possible to avoid rupture only for a certain range of the gap *γ*. If, in particular, conditions are such that the lower chain is stable in isolation, then *γ* can be arbitrarily large. If, alternatively, the lower chain is not stable in isolation, then *γ* must be small enough for the upper chain to stabilize the lower one but not so small that the upper chain ruptures. Figure 8 contains a plot of the critical value *γ*_*c*_ of the gap at which the base configuration loses stability versus the number *N* of balls in each chain for various values of *α*. In particular, for *α*=750, the plot shows that the base configuration is stable for *N*=20 balls if *γ* is less than $\gamma_{c}^{N=20}\approx 4.7$.

From the stability analysis, we obtain the scaling for the asymptotic behaviour of *γ*_*c*_ for N→∞

5.6 $$\gamma_{c}=\tilde{k}{\left(\frac{\alpha }{N}\right)}^{1/3}-1,$$

with $\tilde{k}$ being a constant of order unity.

#### (iii) Sample modes for chains of eight Balls

**Figure 9.**
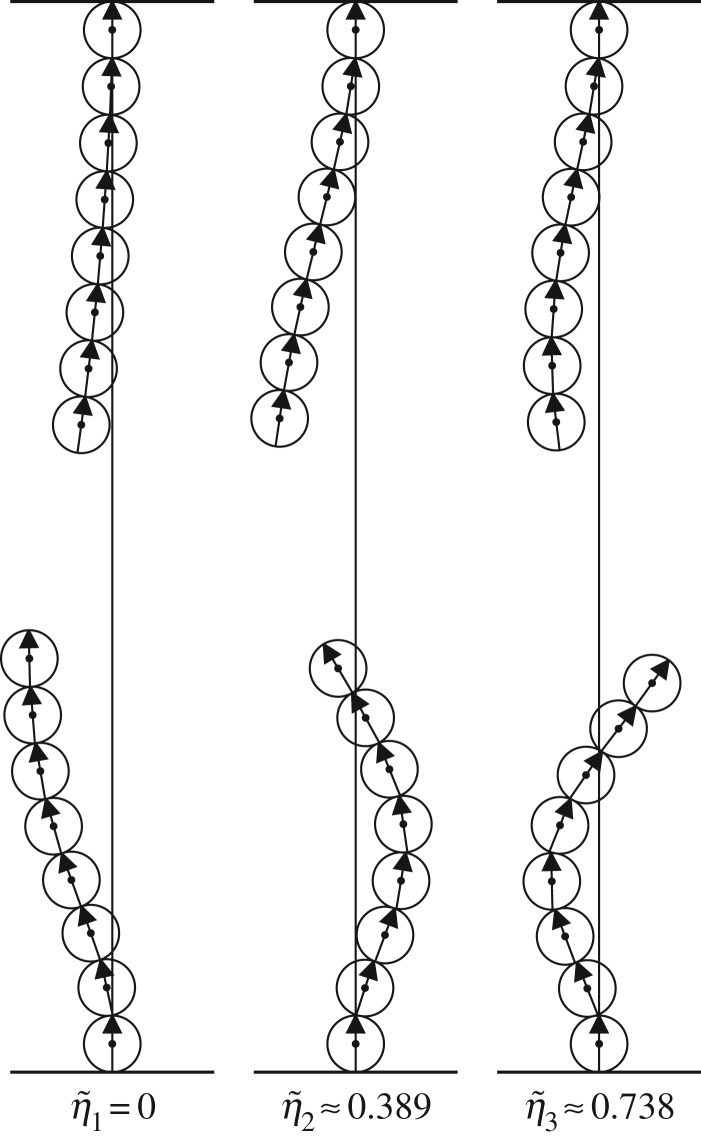
The first three modes ${\tilde{\text{v}}}_{1}$, ${\tilde{\text{v}}}_{2}$ and ${\tilde{\text{v}}}_{3}$ for two clamped chains, each containing eight balls with *α*=75, separated by the critical gap *γ*_*c*_≈2.907 where the first mode destabilizes. The corresponding eigenvalue is indicated below each mode.

The first three modes for a chain of *N*=8 balls with *α*=75 at the critical gap *γ*_*c*_≈2.907 are depicted in figure 9. The first mode is unstable for this combination of parameter values and the corresponding eigenvalue vanishes.

## 6. Two equal free chains

We now consider the consequence of relaxing the boundary conditions to allow the ball at the base of the lower chain to both rotate and slide while the ball at the apex of the upper chain is allowed to rotate but cannot slide. Under these conditions, the lower chain will always topple without the presence of the upper chain. That presence is not, however, sufficient to guarantee that the lower chain will not topple. Moreover, the magnetic force exerted by the upper chain may cause the lower chain to lose contact with the base. We explore this possibility before considering the possibility of stability in the absence of such a lift off.

### (a) Lift off of the lower chain

The threshold for lift off of the lower chain should occur when the magnitude of the attractive magnetic force between the upper chain and the lower chain is balanced by the gravitational force acting on the lower chain. In dimensionless form, this criterion yields a condition analogous to conditions (5.1) and (5.4) that arise in our discussion of rupture:

6.1 $$\alpha \sum_{i=1}^{N}\left[\sum_{k=1}^{N}\frac{1}{{\left(i+k+\gamma -1\right)}^{4}}\right]=N.$$

It is intuitively evident that for given values of *N* and *α*, the critical gap *γ*_*l*_ for the lift off of the lower chain must be less than the critical value *γ*_*c*_ associated with deviations from the coaxial, rectilinear shape of the base configuration.

### (b) General stability threshold

**Figure 10.**
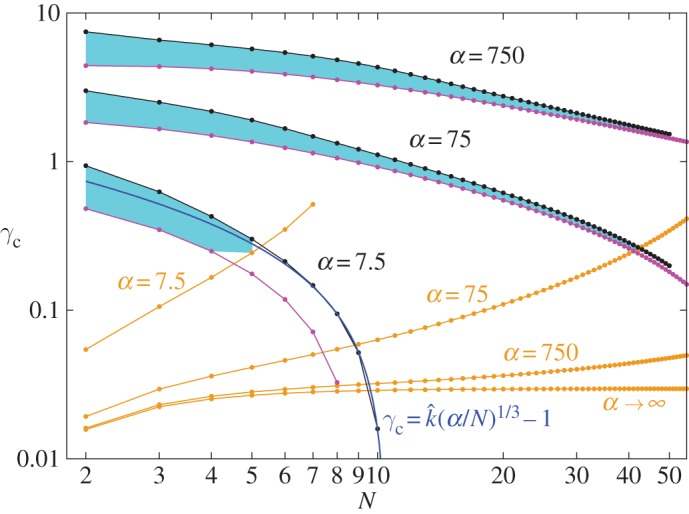
Critical gap *γ*_*c*_ (black) below which two free chains, each containing *N* balls, are stable against buckling of the lower chain. Several curves for different values of *α* are plotted. Orange lines show the value *γ*_*r*_ of *γ* below which the upper chain will rupture (§5a). Magenta lines show the value *γ*_*l*_ of *γ* below which the lower chain will exhibit lift off (§6a). Cyan areas indicate the allowed ranges for *N* and *γ* to have stable chains that neither rupture nor lift off for a given *α*. The blue line exemplarily shows the asymptotic behaviour of *γ*_*c*_ as N→∞.

If the ball at the base of the lower chain is free to slide and rotate and the ball at the apex of the upper chain is free to rotate, the Hessian $\hat{\text{H}}$ of the base configuration is the (4*N*−1)×(4*N*−1) matrix that results on deleting the row and column corresponding to *t*_2*N*_ from (3.15). Figure 10 contains a plot of the critical value *γ*_*c*_ of the gap *γ* at which the base configuration becomes unstable versus the number *N* of balls in each chain for various values of *α*. Owing to the possibility of lift off, the range of feasible gaps diminishes monotonically with increasing *N*. Since the lower chain is never stable without the upper chain, the curves for *γ*_*c*_ extend to *N*=2 for all values of *α*. For *α*=750, the range of *γ*_*c*_ shrinks from $\left[\gamma_{\;l}^{N=2},\gamma_{c}^{N=2}]=[4.42,7.47\right]$ to $\left[\gamma_{l}^{N=50},\gamma_{c}^{N=50}]=[1.44,1.53\right]$. The curves appearing in figure 10 that depict the critical value *γ*_ *r*_ of *γ* associated with the rupture of the upper chain at the junction between its two uppermost balls are identical to those contained in figure 8. The results show that the values of *N* and *α* can combine to decide whether the upper chain ruptures or the lower chain lifts off. If, for example, *α*=7.5 and *N*=5, then rupture occurs at a value of *γ* higher than that required to induce lift off.

The scaling of *γ*_*c*_ for N→∞ differs from that appearing in (5.6) only to the extent that the prefactor $\hat{k}$ takes a different value. Importantly, that value is also of order unity.

### (c) Sample modes for chains of eight balls

**Figure 11.**
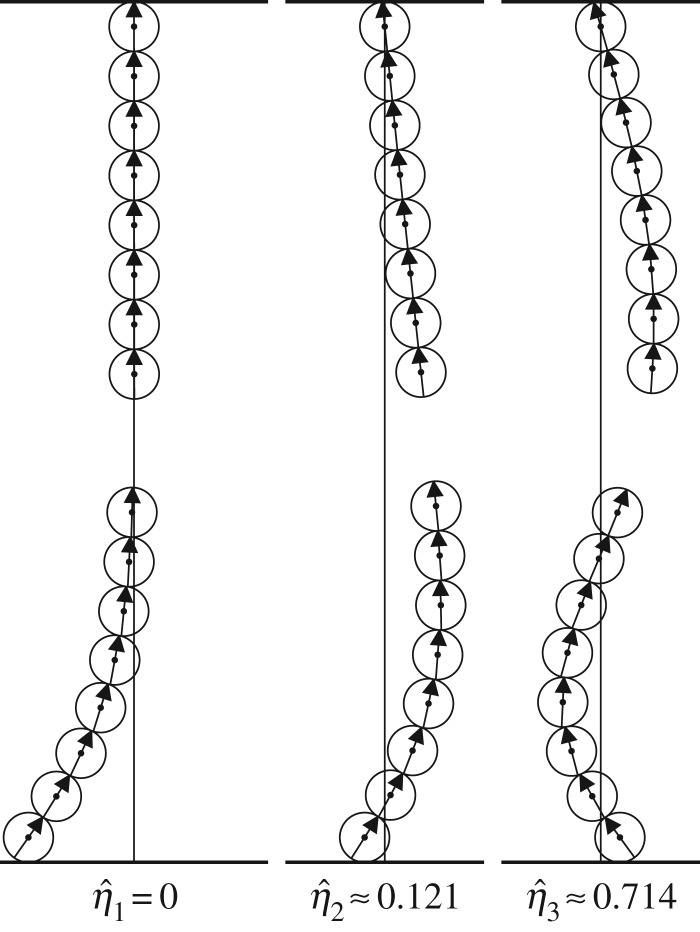
The first three modes ${\hat{\text{v}}}_{1}$, ${\hat{\text{v}}}_{2}$ and ${\hat{\text{v}}}_{3}$ for two free chains, each containing eight balls with *α*=75, separated by the critical gap *γ*_*c*_≈1.330 where the first mode destabilizes. The corresponding eigenvalue is indicated below each mode.

The first three modes for a chain of *N*=8 balls with *α*=75 at the critical gap *γ*_*c*_≈1.330 are depicted in figure 11. The first mode is unstable for this combination of parameters and the corresponding eigenvalue vanishes. It is interesting to observe that for the first mode (leftmost panel in figure 11), the upper chain is very nearly straight and, thus, remains very close to its base configuration. For that first mode, the lowest ball in the upper chain has the largest horizontal deflection (to the right) of all balls in the upper chain, which is only about 0.5% of the diameter of a single ball.

## 7. Chains with opposing dipole orientations

We now briefly consider a class of base configurations in which the dipole moments are arranged so that the chains interact repulsively. Specifically, we suppose that the dipole moments of the balls contained in the upper chain point downward while the balls in the lower chain point upward.

For sufficiently large values of the parameter *α* and the gap *γ*, the effective bending stiffness of the chains is enough to ensure that the base configuration is stable. However, the base configuration is unstable below a certain critical value *γ*_*c*_ of *γ* depending on *N* and *α*. As in the study of a single clamped chain presented in §4, it is crucial that the ball at the base of the lower chain be clamped. Otherwise the system is unconditionally unstable. Importantly, however, no analogous restriction exists for the upper chain and we will consider situations in which the ball at the apex of that chain is either clamped or fixed horizontally while being allowed to rotate.

Before presenting results, we summarize the most salient differences in the formulation that ensue if the dipole moments are arranged as explained above. First, (3.10) is replaced by

7.1 $${\text{n}}_{i}=\left\{ \begin{array}{ll} s_{i}{\text{e}}_{1}+{\text{e}}_{2}, & i=1,\ldots ,N,\\ s_{i}{\text{e}}_{1}-{\text{e}}_{2} & i=N+1,\ldots ,2N. \end{array}\right.$$

Second, although the Hessian still admits a decomposition of the form (3.15), the magnetic contribution $\overset{ˇ}{\text{A}}$ is indefinite instead of positive-semidefinite. This is in keeping with the intuitive expectation that repulsive magnetic interactions can also destabilize the system independent of gravity. Moreover, $\overset{ˇ}{\text{A}}$ takes the form

7.2 $$\overset{ˇ}{\text{A}}={\overset{ˇ}{\text{A}}}_{0}+\sum_{n=1}^{2N-1}\sum_{l=0}^{4}\frac{{\overset{ˇ}{\text{A}}}_{ln}\gamma^{i}}{{\left(\gamma +n\right)}^{7}}$$

and thus depends on *γ* at different orders. Here, ${\overset{ˇ}{\text{A}}}_{0}$ and ${\overset{ˇ}{\text{A}}}_{ln}$ are 10*N*−4 coefficient matrices with rational entries depending only on *N* for each combination of *l*=0, 1, 2, 3, 4 and *n*=1,…,2*N*−1.

### (a) General stability threshold

**Figure 12.**
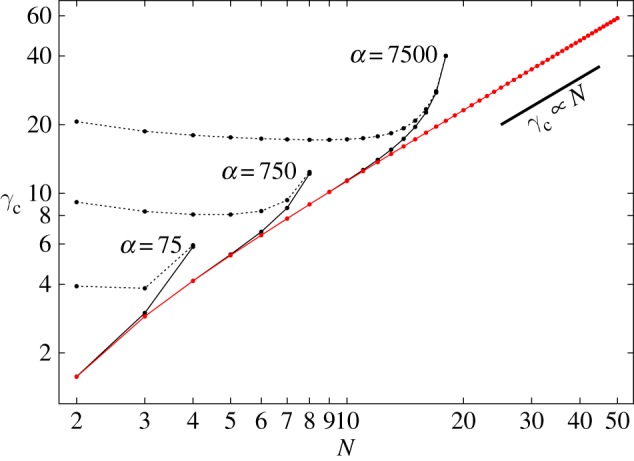
Critical gap *γ*_*c*_ (black) above which two chains (each containing *N* balls) with opposing dipole orientations are stable. Several curves for different values of *α* are included in the plot. While dashed lines refer to situations where the ball at the apex of the upper chain can rotate, the solid lines pertain to situations where that ball is clamped. The red line depicts the limit α→∞ for the clamped upper chain and the thick black line segment shows the respective asymptotic slope for *N*≫1.

Figure 12 contains a plot of the critical value *γ*_*c*_ of the gap at which the base configuration loses stability versus the number *N* of balls in each chain for various values of *α*.

If both chains are clamped, then *γ*_*c*_ is bounded below as α→∞. In this limit, the base configuration is symmetric (again with respect to the line through the midpoint of the gap and orthogonal to the chains). Applying the additional limit of *N*≫1, we find a linear dependence of *γ*_*c*_ on *N* with a prefactor of order unity (*γ*_*c*_≈1.172*N*). It can therefore be reasoned that the base configuration is stable only if the chains are separated by a gap no less than their length. The collection of stable combinations of *γ* and *N* must lie above the curve provided by *γ*_*c*_(*N*) as α→∞. That such a lower bound exists is intuitively clear, because increasing *α* not only increases the effective bending stiffness of the chains but also increases the repulsive forces between the chains. Whereas the first of these effects is stabilizing, the second is destabilizing.

If the upper chain is not clamped, the threshold for stability decreases relative to the clamped case and the stability is lost at a larger value of the gap. The difference is more pronounced for small values of *N* and large values of *α*. Furthermore, *γ*_*c*_(*N*) has a global minimum for given *α* (dashed lines in figure 12). For decreasing *N*, however, *γ*_*c*_ is again increasing because with fewer balls the upper chain can overcome gravity more easily and rotate away from vertical.

### (b) Sample modes for chains of six balls

**Figure 13.**
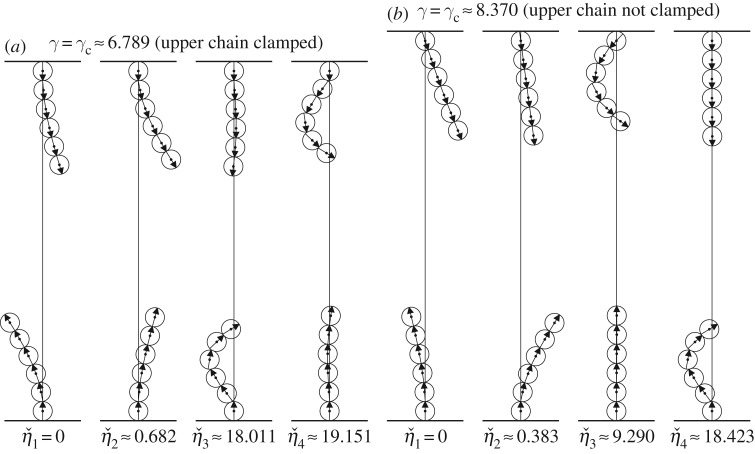
The first four modes ${\overset{ˇ}{\text{v}}}_{1}$, ${\overset{ˇ}{\text{v}}}_{2}$, ${\overset{ˇ}{\text{v}}}_{3}$ and ${\overset{ˇ}{\text{v}}}_{4}$ for two chains, each containing six balls with *α*=750, separated by the critical gap *γ*_*c*_≈6.789 (*a*) and *γ*_*c*_≈8.370 (*b*) where the first mode becomes unstable. In (*a*), both chains are clamped. In (*b*), only the lower chain is clamped and the upper is fixed and allowed to rotate at its apex. The corresponding eigenvalue is indicated below each mode.

Figure 13 contains the first four modes of the system with *N*=6 for *α*=750 at the critical gap *γ*_*c*_, for situations in which both chains are clamped and in which the lower chain is clamped while the upper chain is fixed horizontally with the ball at its apex being allowed to rotate.

## 8. Discussion

We investigated the linear stability of two interacting chains of magnetic balls, described as constrained mechanisms, subject to gravitational and magnetic forces. Our formulation yields the entries of the Hessian of the base configuration in exact form. With the aid of a symbolic manipulation programme such as Mathematica, we obtained the eigenvalues and (eigen)modes of that Hessian up to machine precision. We found that the space of essential parameters *N* (number of balls), *γ* (dimensionless gap size) and *α* (strength of magnetic forces relative to gravity) has a rich structure with stable and unstable domains. That structure depends only on the applied boundary conditions for the endpoints of the chains which are in contact with the ground and the ceiling. For *N* sufficiently large, we confirmed previous analyses showing that a single vertical chain of balls clamped at its base behaves like a classical heavy elastica cantilevered at its base. This supports the notion of an effective bending stiffness induced by the magnetic interactions for this system. Furthermore, we discovered two distinct families of (eigen)modes. While one family is conditionally stable, meaning that the modes lose stability for some non-zero values of *α*, the other family is unconditionally stable and its modes destabilize only if the magnetic interactions are fully switched off (*α*=0). This can be viewed as a degeneracy that arises because the gravitational energy of the system does not depend on the degrees of freedom corresponding to the orientations of the magnetic dipole moments. The stability threshold for the buckling of the single chain was found to agree well with some simple experimental tests. For two chains separated by a finite gap, we encountered two instabilities distinct from the buckling instability of the lower chain that involve changes in the topology of the system. The upper chain can rupture due to gravity and/or the attractive force exerted by the lower chain. Moreover, the lower chain, if not fixed at its base, can lift off due to the attractive force exerted by the upper chain. These additional instabilities can considerably reduce the sets of stable parameter combinations.

While we treated only problems involving chains with equal numbers of balls, our method is immediately applicable to chains with different numbers of balls. Moreover, with a minor extension, our approach can be used to treat chains consisting of balls of different sizes. A potentially limiting feature of our model arises from neglecting the frictional forces that act between the balls and which might, at least under certain circumstances, act to stabilize chains against shape changes. A first step towards incorporating these potentially important effects would be to allow for frictionless rolling contact between the balls while neglecting sliding contact. Such a generalization would couple the positional and orientational degrees of freedom while reducing by half the total number of degrees of freedom. Another possible approach would involve including static friction. However, this would make it necessary to consider discontinuous bifurcations, which fall outside the scope of our current approach.

**Figure 14.**
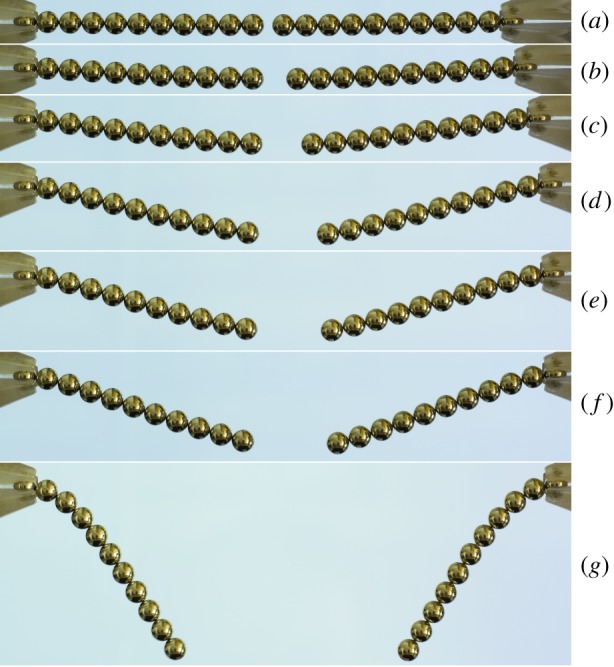
Stability of two horizontal clamped chains separated by a finite gap. Each chain contains 11 balls of diameter 5 mm, mass 0.5 g and magnetic flux density 1.19 T. In (*a*), the chains are nearly horizontal and are separated by approximately 1.7 mm and the distance between the centres of the two clamped balls is approximately 106.7 mm. In (*b*–*h*), the grip on the left is held fixed and the grip on the right is progressively moved to the right. The approximate distances between the centres of the two clamped balls are (*b*) 109.7, (*c*) 112.7, (*d*) 115.1, (*e*) 115.6, (*f*) 116.1 and (*g*) 116.3 mm.

**Figure 15.**
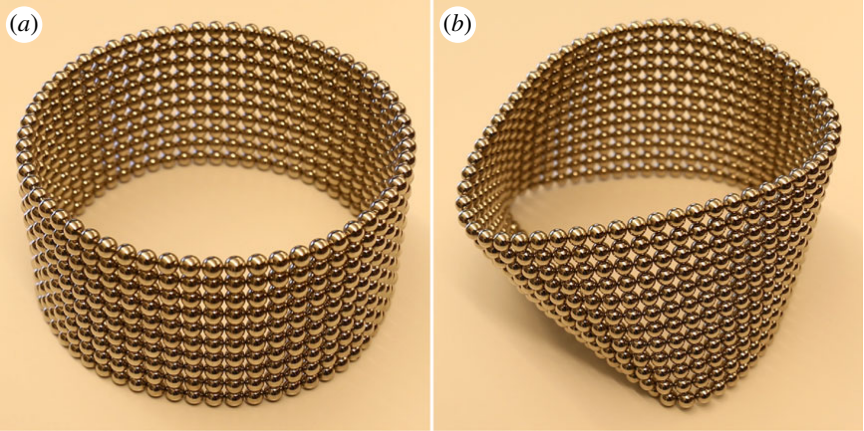
Stability of cylindrical tubes made from square packings of magnetic balls of diameter 5 mm, mass 0.5 g and magnetic flux density 1.19 T. Each tube is formed by stacking circular rings consisting of 60 balls. While a stack of 10 such rings is stable (*a*), a stack of 12 such rings buckles under the influence of gravity (*b*).

Many other possible equilibrium configurations involving assemblies of magnetic balls could be studied for their stability properties. One simple generalization of the configuration studied in this paper arises on rotating the vertical chains by 90^°^, so that their ends are attached to vertical walls and form a shape reminiscent of the catenary curve of a classical heavy chain supported at the end points (figure 14). Under such circumstances, the equilibrium configuration is generally curved and its description would almost certainly need to be determined numerically, preventing the possibility of calculating the energy and, hence, the Hessian in exact form. Another fascinating problem involves cylindrical tubes made from sheets of magnetic balls packed and oriented in various ways. In analogy to the single vertical chain, there should be critical parameter values at which such cylinders buckle under the influence of gravity (figure 15). A minimal example of such a cylinder consists of a configuration in which eight balls sit at the corners of a cube. It would be fascinating to investigate the stability of such a cubic configuration because, as Schönke *et al.* [15] recently demonstrated, its ground state is known to be energetically degenerate with a continuum of possible dipole orientations. What happens to the continuum of states at a bifurcation point?

The sheet made from magnetic balls that is shown in figure 15 has a square packing and can therefore be locally deformed in numerous ways. For example, it can be sheared without rupturing the connections between the balls. A hexagonal packing in which each interior ball has six neighbours provides a fundamentally different way to pack the balls in a sheet. Maintaining the connections between the balls for such a sheet corresponds to a system which is locally incapable of extension or contraction and thus serves as a discrete model for approximately two-dimensional locally unstretchable materials like paper. Owing to the much reduced number of degrees of freedom of such assemblies, a cylinder with hexagonal packing is much more stable against buckling than is a cylinder with square packing. We therefore have the opportunity to investigate the structural stability of approximately two-dimensional materials with different properties. Since there is always a local orientation of the dipoles in a sheet made from magnetic balls, we even have a macroscopic model system in which material anisotropy plays a role. The local bending stiffness, for example, should depend on direction. Moreover, if tearing occurs, it will occur preferentially in a direction parallel to the dipole direction of the magnetic balls.

Beyond the linear, infinitesimal analysis presented here, an important next step would be to conduct a finite amplitude analysis of the system, or even more generally, full dynamical simulations. This would allow for studies of the dynamical processes stemming from arbitrary initial conditions. Moreover, for time-dependent periodic boundary conditions, investigations of interesting phenomena like standing waves and of the frequency response of the system could be performed. For certain parameter regimes, it seems reasonable to expect chaotic behaviour from these dynamical systems.

## Data accessibility

All data are contained within the text.

## Appendix A. Structure of the determinant of the Hessian for a chain clamped at its base

In this appendix, we establish the relation

A 1 $$\det({\bar{\text{A}}}_{0}^{-1}\bar{\text{C}}+\alpha {\text{I}}_{2(N-1)})=\alpha^{N-1}\det[{\left({\bar{\text{A}}}_{0}^{-1}\right)}_{tt}{\bar{\text{C}}}_{tt}+\alpha {\text{I}}_{N-1}]$$

and prove that P(α)=0 has only real and positive roots in *α*.

Since ${\bar{\text{A}}}_{0}^{-1}$ and $\bar{\text{C}}$ have block structures

A 2 $${\bar{\text{A}}}_{0}^{-1}=\left(\begin{array}{cc} {\left({\bar{\text{A}}}_{0}^{-1}\right)}_{tt} & {\left({\bar{\text{A}}}_{0}^{-1}\right)}_{ts}\\ {\left({\bar{\text{A}}}_{0}^{-1}\right)}_{st} & {\left({\bar{\text{A}}}_{0}^{-1}\right)}_{ss} \end{array}\right)\;\text{and}\;\bar{\text{C}}=\left(\begin{array}{cc} {\bar{\text{C}}}_{tt} & \text{0}\\ \text{0} & \text{0} \end{array}\right),$$

with each block being a (*N*−1)×(*N*−1) matrix, their product is given by

A 3 $${\bar{\text{A}}}_{0}^{-1}\bar{\text{C}}=\left(\begin{array}{cc} {\left({\bar{\text{A}}}_{0}^{-1}\right)}_{tt}{\bar{\text{C}}}_{tt} & \text{0}\\ {\left({\bar{\text{A}}}_{0}^{-1}\right)}_{st}{\bar{\text{C}}}_{tt} & \text{0} \end{array}\right)=:\left(\begin{array}{cc} -\bar{\text{B}} & \text{0}\\ -{\bar{\text{B}}}^{\prime } & \text{0} \end{array}\right).$$

It therefore follows from (A 1) that

A 4 $$\det({\bar{\text{A}}}_{0}^{-1}\bar{\text{C}}+\alpha {\text{I}}_{2(N-1)})=\det\left(\begin{array}{cc} -\bar{\text{B}}+\alpha {\text{I}}_{N-1} & \text{0}\\ -{\bar{\text{B}}}^{\prime } & \alpha {\text{I}}_{N-1} \end{array}\right).$$

We now expand the determinant along each of the *N*−1 columns starting from column *N* until the last column 2(*N*−1) of the matrix in (A 4). Each column gives another factor of *α* times the determinant of the reduced matrix which results from removing the corresponding column and the row of the same index, leading finally to

A 5 $$\det({\bar{\text{A}}}_{0}^{-1}\bar{\text{C}}+\alpha {\text{I}}_{2(N-1)})=\alpha^{N-1}\det(-\bar{\text{B}}+\alpha {\text{I}}_{N-1}),$$

which is (A 1).

Since ${\bar{\text{A}}}_{0}$ is positive-definite, so is ${\bar{\text{A}}}_{0}^{-1}$. Thus, by Sylvester's criterion, ${\left({\bar{\text{A}}}_{0}^{-1}\right)}_{tt}$ is also positive-definite. Because ${\bar{\text{C}}}_{tt}$ is negative-definite, it follows from Sylvester's law of inertia that $\bar{\text{B}}=-{\left({\bar{\text{A}}}_{0}^{-1}\right)}_{tt}{\bar{\text{C}}}_{tt}$ has only real and positive eigenvalues regardless of whether $\bar{\text{B}}$ is symmetric (which it is not for *N*>2).

## Appendix B. Critical behaviour of two clamped chains each consisting of two balls

For a pair of clamped chains, each consisting of *N*=2 balls, separated by a gap *γ* (§5), it is possible to obtain an explicit relation for the critical value *α*_*c*_ of *α* at which the position of the lower chain loses stability. That relation has the form

B 1 $$\alpha_{c}^{N=2}(\gamma )=2{\left(2+\gamma \right)}^{5}\sqrt{\frac{{\left(1+\gamma \right)}^{9}\sum_{i=0}^{12}a_{i}\gamma^{i}}{\sum_{j=0}^{31}b_{i}\gamma^{i}}},$$

where the coefficients *a*_*i*_, *i*=0,…,12 and *b*_*i*_, *i*=0,…,31 are given by

**Table:**

*i*	*a*_*i*_	*b*_*i*_	*i*	*b*_*i*_
0	1 092	44 623 017	16	236 359 277 059
1	6 744	622 228 431	17	121 855 281 861
2	20 364	4 275 119 481	18	56 547 295 271
3	39 280	19 240 414 007	19	23 558 396 425
4	53 448	63 645 780 965	20	8 773 187 011
5	53 676	164 687 108 579	21	2 902 235 365
6	40 507	346 552 984 093	22	845 788 851
7	22 968	608 997 759 203	23	214 853 101
8	9 636	911 043 297 553	24	46 945 991
9	2 896	1 177 182 472 519	25	8 676 897
10	588	1 328 723 896 453	26	1 327 627
11	72	1 321 880 254 507	27	163 365
12	4	1 167 341 129 409	28	15 511
13		920 178 840 607	29	1 065
14		650 210 370 673	30	47
15		413 082 353 055	31	1

Consistent with the results of §4a, it follows easily from (B 1) that $\alpha_{c}^{N=2}(\gamma )\to 4=\alpha_{c}^{N=2}$ as γ→∞.
